# Genetic polymorphisms in *ALDH2* are associated with drug addiction in a Chinese Han population

**DOI:** 10.18632/oncotarget.14354

**Published:** 2016-12-29

**Authors:** Chan Zhang, Heng Ding, Yujing Cheng, Wanlu Chen, Qi Li, Qing Li, Run Dai, Manlin Luo

**Affiliations:** ^1^ Department of Blood Transfusion, The First People's Hospital of Yunnan Province, The Affiliated Hospital of Kunming University of Science and Technology, Kunming 650032, Yunnan, China; ^2^ Honghe Center Blood Station, Mengzi 661100, Yunnan, China; ^3^ Department of Blood Transfusion, The Second People's Hospital of Yunnan Province, Kunming 650032, Yunnan, China

**Keywords:** drug addiction, ALDH2, single nucleotide polymorphisms (SNPs), case-control study, association study

## Abstract

We investigated the association between single nucleotide polymorphisms (SNPs) in *ALDH2*, which has been associated with alcohol dependence and several types of diseases, and the risk of drug addiction in a Chinese Han population. In a case-control study that included 692 cases and 700 healthy controls, eight SNPs in *ALDH2* were selected and genotyped using the Sequenom MassARRAY platform. Odds ratios (ORs) and 95% confidence intervals (CIs) were calculated using unconditional logistic regression after adjusting for age and gender. We determined that rs671 is significantly associated with a 1.551-fold increased drug addiction risk (95% CI = 1.263-1.903; *p* < 0.001). In the genetic model analysis, we found that rs671 is associated with an increased risk of drug addiction under additive, dominant and recessive models (*p* < 0.001), while rs886205, rs441 and rs4646778 displayed a decreased drug addiction risk under additive and recessive model, respectively (*p* < 0.05). SNP rs671 remained significant after Bonferroni correction (*p*<0.00125). Additionally, we observed that haplotype “GTCAC” was associated with increased drug addiction risk (OR = 1.668; 95% CI, 1.328–2.094, *p* < 0.001); in contrast, “ATCGC” was a protective haplotype for drug addiction risk (OR = 0.444; 95% CI, 0.281–0.704, *p* < 0.001). Our findings showed that *ALDH2* polymorphisms are significantly associated with the risk of drug addiction in the Chinese Han population.

## INTRODUCTION

Drug addiction is widespread in the world. The number of drug addicts is increasing every year. Previous study have suggested that the drug addiction is a multifactorial process which is influenced by many factors, such as impulsivity, risk taking, anxiety, depression and stress responsivity as well as gene variation [1, 2]. Among these factors, environment factors and curiosity were considered as the most important reason for initial drug use; however, recent study shown that the contribution of inherited factors (mainly genetic) to development of drug addictions is approximately 50% [3].

Aldehyde dehydrogenase 2 (ALDH2) is a key enzyme in the oxidation process of acetaldehyde to acetate. Genome wide association studies have demonstrated that genetic polymorphisms in *ALDH2* are associated with many alcohol-related conditions [4–6], which has led to studies of the association between *ALDH2* and various cancers and disease. For example, rs671 in *ALDH2* was found to be associated with accelerated deterioration of bone marrow in Japanese anemia patients [7]. Rs886205 in *ALDH2* has been identified as a risk marker for esophagus cancer in African populations [8]. However, few studies have investigated the association between genetic polymorphisms in *ALDH2* and the risk of drug addiction.

To investigate the association between *ALDH2* and drug addiction risk, we genotyped eight variants in *ALDH2* associated with alcohol dependence and performed a comprehensive association analysis to identify SNPs associated with drug addiction risk in a Chinese Han population.

## RESULTS

This study included 692 drug addicts (594 men, 98 women; mean age 44.66±6.12 years) and 700 healthy controls (393 men, 307 women; mean age 48.53±9.44 years). The clinical characteristics of cases and controls are shown in Table 1. Age (*p*<0.001) and sex (*p*<0.001) were significantly different between cases and healthy controls. Multivariate analyses were adjusted for age and sex.

**Table 1:** Characteristics of cases and controls in this study

Variables	Case (N=692)	Control (N=700)	Total	*p*-value
Sex, No.(%)				< 0.001^a^
Male	594 (85.8)	393 (56.1)	987(70.9)	
Female	98 (14.2)	307 (43.9)	405(29.1)	
Mean age ±SD	44.66±6.12	48.53±9.44		< 0.001^b^

a *P* values was calculated from Pearson's chi-square tests.

b *P* values was calculated by Welch's t tests.

The minor allele frequencies (MAFs) of the analyzed SNPs in the case and control groups are shown in Table 2. All SNPs were in Hardy-Weinberg equilibrium (HWE) in the controls (*p* > 0.05) with the exception of rs2238152, rs4648328 and rs7296651, which were excluded from subsequent analyses. Using chi-square tests, we determined that rs671 was significantly associated with a 1.551-fold increased drug addiction risk (95% CI = 1.263-1.903; *p* < 0.001), which remained significant after Bonferroni correction (*p*<0.00125).

**Table 2:** Allele frequencies in cases and controls and odds ratio estimates for drug addiction

SNP ID	Gene	Band	Alleles A^a^/B	MAF		HWE *p*-value	ORs	95% CI	*p*-value
				Case	Control				
rs886205	*ALDH2*	12q24.12	A/G	0.118	0.140	0.084	0.825	0.661-1.031	0.091
rs2238152	*ALDH2*	12q24.12	T/G	0.278	0.282	0.024^#^	0.980	0.830-1.157	0.813
rs4648328	*ALDH2*	12q24.12	T/C	0.277	0.285	0.041^#^	0.963	0.817-1.136	0.658
rs441	*ALDH2*	12q24.12	C/T	0.277	0.284	0.052	0.966	0.819-1.141	0.689
rs4646778	*ALDH2*	12q24.12	A/C	0.278	0.284	0.051	0.970	0.822-1.145	0.720
rs671	*ALDH2*	12q24.12	A/G	0.190	0.131	0.619	1.551	1.263-1.903	< 0.001*
rs11066028	*ALDH2*	12q24.12	A/C	0.095	0.094	0.501	1.013	0.785-1.036	0.922
rs7296651	*ALDH2*	12q24.12	C/G	0.121	0.144	0.021^#^	0.814	0.653-1.014	0.066

MAF, minor allelic frequency; HWE, Hardy-Weinberg Equilibrium; ORs, odds ratios; CI: confidence interval.

a Minor allele;

# HWE *p*-value ≤ 0.05 was excluded;

* *p* value ≤ 0.05.

Bonferroni's multiple adjustment was applied, with *p*<0.00125 (0.05/40).

The genotype frequencies of the *ALDH2* polymorphisms were shown in Table 3. Compared with the “CC” genotype, the “AC” frequency of rs4646778 polymorphism among cases were significantly different from the controls (AC vs. CC: OR= 1.271, 95% CI = 1.001-1.614; *p* = 0.048), which suggested that the rs4646778 polymorphism had an increased effect on drug addiction risk. Similarly, compared with individuals with the rs671 “GG” genotype, individuals with “AA” genotype had a significantly increased drug addiction risk (AA vs. GG: OR= 2.638, 95% CI = 1.180-5.899; *p* = 0.018). In addition, individuals with rs671 “AG” genotype also had a significantly increased drug addiction risk compared with “GG” genotype (AG vs. GG: OR= 1.646, 95% CI = 1.269-2.133; *p* < 0.001), which remained significant after Bonferroni correction (*p*<0.00125).

**Table 3:** Genotypes distribution of SNPs and their associations with the risk of drug addiction

SNP ID	Alleles A^a^/B	genotype	No. (frequency)		Logistic regression	
			Case (%)	Control (%)	OR(95% CI)	*p*^a^
rs886205	A/G	GG	537(77.6)	512(73.1)	1	
		AA	9(1.3)	8(1.1)	0.696(0.255-1.902)	0.479
		AG	146(21.1)	180(25.8)	0.770(0.587-1.008)	0.057
rs441	C/T	TT	347(50.1)	369(52.7)	1	
		CC	39(5.7)	67(9.6)	0.655(0.414-1.036)	0.070
		CT	306(44.2)	264(37.7)	1.262(0.994-1.602)	0.056
rs4646778	A/C	CC	346(50.1)	369(52.8)	1	
		AA	39(5.6)	67(9.6)	0.657(0.415-1.040)	0.073
		AC	306(44.3)	263(37.6)	1.271(1.001-1.614)	0.048*
rs671	A/G	GG	452(65.3)	526(75.1)	1	
		AA	23(3.3)	10(1.4)	2.638(1.180-5.899)	0.018*
		AG	217(31.4)	164(23.5)	1.646(1.269-2.133)	< 0.001*
rs11066028	A/C	CC	568(82.1)	573(81.9)	1	
		AA	7(1.0)	4(0.6)	1.409(0.392-5.509)	0.569
		AC	117(16.9)	123(17.5)	0.935(0.692-1.263)	0.662

^a^ Minor alleles, OR: Odds ratio, CI: Confidence interval;

a *p* value were calculated by unconditional logistic regression adjusted by gender and age;

* *p* ≤ 0.05; Bonferroni's multiple adjustment was applied, with *p*<0.00125.

We further assessed the association between each SNP and drug addiction risk in an unconditional logistic regression analysis, which was performed using three models: additive, dominant and recessive model (Table 4). The minor allele A of rs886205 was associated with a decreased drug addiction risk under additive model (OR= 0.782, 95% CI = 0.613-0.998; *p* = 0.048). The minor alleles C of rs441 and A of rs4646778 also displayed significantly decreased drug addiction risk under recessive model (rs441: OR= 0.591, 95% CI = 0.378-0.924; *p* = 0.021; rs4646778: OR= 0.592, 95% CI = 0.378-0.923; *p* = 0.021). Additionally, the minor allele A of rs671 displayed a remarkable increased drug addiction risk under additive model (OR= 1.640, 95% CI = 1.307-2.057; *p* < 0.001), dominant model (OR= 1.706, 95% CI = 1.325-2.196; *p* < 0.001) and recessive model (OR= 2.293, 95% CI = 1.030-2.106; *p* = 0.017). However, only rs671 remained significant in the additive and dominant model after Bonferroni correction (*p*<0.00125).

**Table 4:** Association between SNPs and drug addiction in multiple inheritance models

SNP ID	Minor allele	Additive model			Dominant model			Recessive model		
		OR	95% CI	*p*-value	OR	95% CI	*p*-value	OR	95% CI	*p*-value
rs886205	A	0.782	0.613-0.998	0.048*	0.765	0.587-0.997	0.047	0.740	0.271-2.018	0.556
rs441	C	0.996	0.831-1.195	0.969	1.144	0.911-1.437	0.247	0.591	0.378-0.924	0.021*
rs4646778	A	1.000	0.834-1.200	0.996	1.151	0.917-1.446	0.226	0.592	0.378-0.923	0.021*
rs671	A	1.640	1.307-2.057	< 0.001*	1.706	1.325-2.196	< 0.001*	2.293	1.030-2.106	0.042*
rs11066028	A	0.977	0.741-1.287	0.867	0.953	0.710-1.281	0.752	1.486	0.397-5.568	0.556

ORs, odds ratios; CI: confidence interval.

* *p* value ≤ 0.05; Bonferroni's multiple adjustment was applied, with *p*<0.00125.

**Figure 1:**
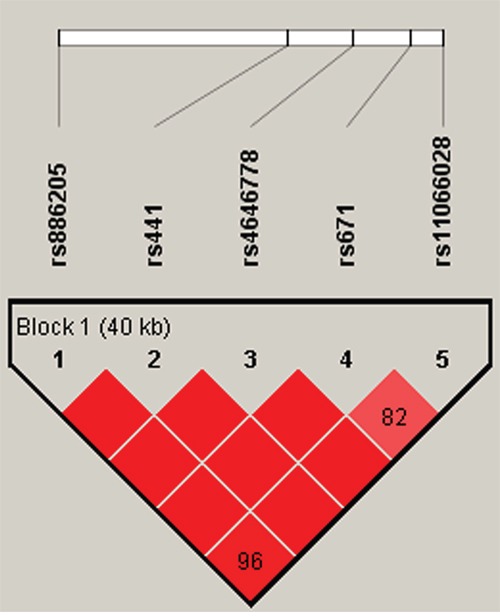
Haplotype block map for all the SNPs of the ALDH2 gene

We further characterized the SNPs in *ALDH2* SNPs using linkage disequilibrium (LD) and haplotype analyses. In the control group, one LD block was detected (Figure 1). This block consisted of 5 closely linked SNPs, rs886205, rs441, rs4646778, rs671 and rs11066028. Finally, a haplotype-based association study was performed to show the associations between *ALDH2* haplotypes and drug addiction risk (Table 5). The haplotype “GTCAC” was associated with increased drug addiction risk (OR = 1.668; 95% CI, 1.328–2.094, *p* < 0.001); in contrast, haplotype “ATCGC” was associated with decreased drug addiction risk (OR = 0.444; 95% CI, 0.281–0.704, *p* < 0.001). Both of the haplotypes remained significant after Bonferroni correction (*p*<0.00125).

**Table 5:** *ALDH2* haplotype frequencies and the association with drug addiction risk

Block ID	Haplotype	Freq(case)	Freq(control)	χ2	Pearson's *p*	OR	95% CI		*p*-adj
1	ATCGA	0.095	0.091	0.128	0.7201	0.999	0.756	1.320	0.996
	GTCAC	0.190	0.129	19.65	< 0.001*	1.668	1.328	2.094	< 0.001*
	GCAGC	0.278	0.283	0.101	0.7511	1.003	0.836	1.202	0.977
	ATCGC	0.024	0.049	12.76	< 0.001*	0.444	0.281	0.704	< 0.001*
	GTCGC	0.414	0.445	2.726	0.0987	0.859	0.731	1.009	0.065

OR: odd ratio; CI: confidence interval.

*p*-adj: *p*-value was adjusted by sex and age;

* *p* value ≤ 0.05; Bonferroni's multiple adjustment was applied, with *p*<0.00125.

## DISCUSSION

In this study, we investigated the associations between eight selected *ALDH2* SNPs and risk of drug addiction in the Chinese Han population. We found that rs671 is significantly associated with an increased risk of drug addiction, while rs886205, rs441 and rs4646778 displayed decreased drug addiction risk. Our results suggest that the polymorphisms of *ALDH2* may play an important role in the risk of drug addiction in the Chinese Han population.

The *ALDH2* gene is located on chromosome 12. Rs671 (Glu504Lys) is a functional SNP in *ALDH2*, which could decrease the normal dehydrogenase activity of ALDH2 by approximately 90% [9, 10]. Previous study has shown that rs671 is associated with the risk of upper aerodigestive tract cancers in a Japanese population [11]. Later, Chinese study shown that individuals with the rs671 A allele may experience increased coronary heart disease risk because of interfering HDL-C and endothelial ADMA concentrations [12]. Recently, another study demonstrated that rs671 polymorphism may influence post-stroke epilepsy susceptibility by affecting plasma 4-HNE levels [13]. In our study, we found that “AG” and “AA” genotypes of rs671 are significantly associated with an increased risk of drug addiction. As a result, we concluded that carriers of the allele “A” of *ALDH2* rs671 may have low activity of ALDH2 enzymes and reduced metabolism, resulting in increased plasma concentration and delayed clearance of drugs. As far as we know, our study is the first to demonstrate that rs671 affect the development of drug addiction. Therefore, further research with a larger sample size is needed to confirm our data.

Rs886205 (A>G) is an SNP in the promoter of *ALDH2*, which has been identified as a risk marker for esophageal squamous cell carcinoma in several Western populations [8]. Furthermore, one recent study has reported that rs886205 is associated with altered methylation levels of the negative regulatory promoter fragment and corresponding ALDH2 protein levels in alcohol-dependent patients’ blood during withdrawal [14]. Our data shown that rs886205 is associated with a decreased risk of drug addiction. We concluded that it may be associated with drug addiction by influencing the adaptation of ALDH2 protein levels during detoxification.

Previous study have reported that rs4646778 polymorphism may influence the methadone dose and adverse reactions in patients with heroin addiction [15]. In our study, we also found that rs4646778 and rs441 displayed a decreased risk of drug addiction. However, literatures about rs4646778 and rs441 are relatively rare. So these results should be confirmed in further studies.

The Bonferroni correction is one of the most important methods used to address false discovery rates resulting from multiple testing. We found that only rs671 remained significant after Bonferroni correction, this may due to our strict SNP filtering criteria and small sample size. Additionally, the Bonferroni correction adjusts the value of alpha based on the number of tests performed, and is thus conservative; in some cases, truly significant differences may be deemed non-significant as a result of type II errors [16].

Our study had several intrinsic limitations. For example, drug addiction is a very complicated process, and environment factors such as level of education and income are important risk factors for drug addiction. Because our study had a relatively small size, and it did not incorporate data regarding education and income, we could not explore the interactions between genetic polymorphisms and environmental factors in drug addicts. Therefore, the relationship between *ALDH2* polymorphisms and environment factors in drug addicts must be evaluated in future studies.

Our present study provided evidence that SNPs in the *ALDH2* are associated with drug addiction in a Chinese Han population. It is possible that these variants are drug addiction risk factors and these data can provide a theoretical foundation for other researchers to further study the association between the *ALDH2* gene and drug addiction risk in the Chinese Han or other populations.

## MATERIALS AND METHODS

### Subjects

In this study, all subjects were restricted to genetically unrelated Han Chinese individuals. A total of 692 unrelated former severe common illicit drug addicts (including opioid, stimulants, and marijuana) were recruited from the First People's Hospital of Yunnan Province from June 2014 to July 2016, and 700 healthy controls were randomly selected from the same hospital. Subjects were at least 18 years old who had one or more years of daily multiple uses of narcotics and underwent repeated random and observed urine tests. All patients had a clinical diagnosis of drug addiction. All psychiatric axis-I disorders were used as exclusion criteria. In addition, participants were excluded if they had a history of a seizure disorder (except cocaine-induced seizures) or severe medical illness. Individuals currently being treated with psychotropic medications or with psychiatric symptoms, including psychosis, dementia, suicidal or homicidal ideation, mania or depression requiring antidepressant therapy were also excluded.

All of the participants signed an informed consent agreement. The Human Research Committee for Approval of Research Involving Human Subjects, the First People's Hospital of Yunnan Province, approved the use of human blood in this study.

### SNP selection and genotyping

Eight SNPs were chosen from previously published polymorphisms associated with alcohol dependence [17–20], with minor allele frequencies >5% in the HapMap Chinese Han Beijing population. DNA was extracted from whole-blood samples by GoldMag-Mini Whole Blood Genomic DNA Purification Kit (GoldMag Co. Ltd. Xi’an City, China). Quantification of the extracted DNA was performed using NanoDrop 2000 (Thermo Scientific, Waltham, Massachusetts, USA). The multiplexed SNP MassEXTENDED assay was designed using Sequenom MassARRAY Assay Design 3.0 Software [21]. Genotyping was done with the Sequenom MassARRAY RS1000 system using the standard protocol recommended by the manufacturer. Primers of PCR which were used for each SNP in our study are listed in Table 6. Data management and analysis was done using Sequenom Typer 4.0 Software [21, 22].

**Table 6:** Primers used for this study

SNP_ID	1st_PCRP	2st_PCRP	UEP_SEQ
rs886205	ACGTTGGATGTCTCG CTTTTGGGTTTACGG	ACGTTGGATGCCTTT GACCCCAATGTGAAC	TGGAGCATCAGCCGGG
rs2238152	ACGTTGGATGTGTTGTA AAAAGCACCAACC	ACGTTGGATGAATC CCACCTTTATTTAAG	CCAACCTCAAAGCCAAA
rs4648328	ACGTTGGATGTTTAG CTTCTGCTCTCTACC	ACGTTGGATGTTTGC TCTGTAAGCTCCACG	TCTCTACCATATCCAGGT
rs441	ACGTTGGATGAGCCTG GGTGCCAGAGAGA	ACGTTGGATGCCCTG ACAGCATTCACTTAG	GTGCCAGAGAGAGACTCGG
rs4646778	ACGTTGGATGTATGCA GGCAACAAGACAAC	ACGTTGGATGGTTTT CTGCTATTGGCCCTG	ACAAGACAACTGGGAAAT
rs671	ACGTTGGATGAGGTC CCACACTCACAGTTT	ACGTTGGATGTTGGT GGCTACAAGATGTCG	ggccgACACTCACAGTTTTCACTT
rs11066028	ACGTTGGATGCCCC ACCATAAAGCTATGAC	ACGTTGGATGTGGAT GAAGTGTACCCACTG	gagaACAGCCAGTCTTGTTT
rs7296651	ACGTTGGATGGGGCA AGACCCAGATTTGAA	ACGTTGGATGCACGT GGCCTGTAACTATGA	ACATCATTGGCCTATAACT

### Statistical analysis

We used Microsoft Excel and the SPSS 18.0 statistical package (SPSS, Chicago, IL, USA) to perform statistical analyses. All *p* values presented in this study are two sided, and *p* = 0.05 was considered the cutoff for statistical significance. The differences in the characteristics of the case and control study population were compared using the chi-squared test (for categorical variables) and Welch's t tests (for continuous variables). In all analyses, the lower frequency allele was considered as the ‘risk’ allele. Control genotype frequencies for each SNP were tested for departure from Hardy-Weinberg equilibrium (HWE) using Fisher's exact test. The χ2 test was used to compare allele and genotype frequencies in cases and controls [23]. In order to assess the association between each genotype and the risk of drug addiction, three models were used, including additive model, dominant model and recessive model. The effects of the polymorphisms on the risk of drug addiction were expressed as odds ratios (ORs) with 95% confidence intervals (95% CIs), computed using unconditional logistic regression analysis with adjustments for age and sex [24]. Finally, the patterns of linkage disequilibrium (LD) and haplotype construction was evaluated by Haploview software package (version 4.2) [25]. All *p* values were Bonferroni corrected, and statistical significance was set at *p*<0.00125 (0.05/40).

## References

[1] Johnson JL, Leff M (1999). Children of substance abusers: overview of research findings. Pediatrics.

[2] Reynolds EK, Magidson J. F., Mayes L. C., Lejuez C. W (2010). Risk-taking behaviors across the transition from adolescence to young adulthood. Young adult mental health.

[3] Kendler KS, Jacobson KC, Prescott CA, Neale MC (2003). Specificity of genetic and environmental risk factors for use and abuse/dependence of cannabis, cocaine, hallucinogens, sedatives, stimulants, and opiates in male twins. The American journal of psychiatry.

[4] Matsuo K, Hamajima N, Shinoda M, Hatooka S, Inoue M, Takezaki T, Tajima K (2001). Gene-environment interaction between an aldehyde dehydrogenase-2 (ALDH2) polymorphism and alcohol consumption for the risk of esophageal cancer. Carcinogenesis.

[5] Yokoyama A, Muramatsu T, Ohmori T, Yokoyama T, Okuyama K, Takahashi H, Hasegawa Y, Higuchi S, Maruyama K, Shirakura K, Ishii H (1998). Alcohol-related cancers and aldehyde dehydrogenase-2 in Japanese alcoholics. Carcinogenesis.

[6] Matsuo K, Wakai K, Hirose K, Ito H, Saito T, Tajima K (2006). Alcohol dehydrogenase 2 His47Arg polymorphism influences drinking habit independently of aldehyde dehydrogenase 2 Glu487Lys polymorphism: analysis of 2,299 Japanese subjects. Cancer epidemiology, biomarkers & prevention.

[7] Hira A, Yabe H, Yoshida K, Okuno Y, Shiraishi Y, Chiba K, Tanaka H, Miyano S, Nakamura J, Kojima S, Ogawa S, Matsuo K, Takata M, Yabe M (2013). Variant ALDH2 is associated with accelerated progression of bone marrow failure in Japanese Fanconi anemia patients. Blood.

[8] Bye H, Prescott NJ, Matejcic M, Rose E, Lewis CM, Parker MI, Mathew CG (2011). Population-specific genetic associations with oesophageal squamous cell carcinoma in South Africa. Carcinogenesis.

[9] Crabb DW, Edenberg HJ, Bosron WF, Li TK (1989). Genotypes for aldehyde dehydrogenase deficiency and alcohol sensitivity. The inactive ALDH2(2) allele is dominant. The Journal of clinical investigation.

[10] Yoshida A, Huang IY, Ikawa M (1984). Molecular abnormality of an inactive aldehyde dehydrogenase variant commonly found in Orientals. Proceedings of the National Academy of Sciences of the United States of America.

[11] Oze I, Matsuo K, Hosono S, Ito H, Kawase T, Watanabe M, Suzuki T, Hatooka S, Yatabe Y, Hasegawa Y, Shinoda M, Tajima K, Tanaka H (2010). Comparison between self-reported facial flushing after alcohol consumption and ALDH2 Glu504Lys polymorphism for risk of upper aerodigestive tract cancer in a Japanese population. Cancer science.

[12] Guo YJ, Chen L, Bai YP, Li L, Sun J, Zhang GG, Yang TL, Xia J, Li YJ, Chen XP (2010). The ALDH2 Glu504Lys polymorphism is associated with coronary artery disease in Han Chinese: Relation with endothelial ADMA levels. Atherosclerosis.

[13] Yang H, Song Z, Yang GP, Zhang BK, Chen M, Wu T, Guo R (2014). The ALDH2 rs671 polymorphism affects post-stroke epilepsy susceptibility and plasma 4-HNE levels. PLoS One.

[14] M Haschemi Nassab, Rhein M, Hagemeier L, Kaeser M, Muschler M, Glahn A, Pich A, Heberlein A, Kornhuber J, Bleich S, Frieling H, Hillemacher T (2016). Impaired Regulation of ALDH2 Protein Expression Revealing a Yet Unknown Epigenetic Impact of rs886205 on Specific Methylation of a Negative Regulatory Promoter Region in Alcohol-Dependent Patients. European addiction research.

[15] Tian JN, Wang SC, Chen CH, Tsou HH, Ho IK, Chan HW, Fang CP, Hsiao CF, Tan HKL, Lin LN, Wu CS, Su LW, Huang CL, Yang YH, Wu JY, Lin KM The genetic polymorphisms in ALDH2 influence the methadone dose and adverse reactions in patients with heroin addiction.

[16] Perneger TV (1998). What's wrong with Bonferroni adjustments. Bmj.

[17] M Haschemi Nassab, Rhein M, Heese P, Glahn A, Frieling H, Linnebank M, Bleich S, Kornhuber J, Heberlein A, Grallert H, Peters A, Rawal R, Strauch K, Hillemacher T (2015). No association between the ALDH2 promoter polymorphism rs886205, alcohol dependence, and risky alcohol consumption in a German population. Psychiatric genetics.

[18] Chang YC, Chiu YF, Lee IT, Ho LT, Hung YJ, Hsiung CA, Quertermous T, Donlon T, Lee WJ, Lee PC, Chen CH, Mochly-Rosen D, Chuang LM (2012). Common ALDH2 genetic variants predict development of hypertension in the SAPPHIRe prospective cohort: gene-environmental interaction with alcohol consumption. BMC cardiovascular disorders.

[19] Dickson PA, James MR, Heath AC, Montgomery GW, Martin NG, Whitfield JB, Birley AJ (2006). Effects of variation at the ALDH2 locus on alcohol metabolism, sensitivity, consumption, and dependence in Europeans. Alcoholism, clinical and experimental research.

[20] Yang H, Zhou Y, Zhou Z, Liu J, Yuan X, Matsuo K, Takezaki T, Tajima K, Cao J (2009). A novel polymorphism rs1329149 of CYP2E1 and a known polymorphism rs671 of ALDH2 of alcohol metabolizing enzymes are associated with colorectal cancer in a southwestern Chinese population. Cancer epidemiology, biomarkers & prevention.

[21] Gabriel S, Ziaugra L, Tabbaa D (2009). SNP genotyping using the Sequenom MassARRAY iPLEX platform. Current protocols in human genetics.

[22] Thomas RK, Baker AC, DeBiasi RM, Winckler W, LaFramboise T, Lin WM, Wang M, Feng W, Zander T, MacConaill LE (2007). High-throughput oncogene mutation profiling in human cancer. Nature genetics.

[23] Adamec C (1964). Example of the use of the nonparametric test. Test X2 for comparison of 2 independent examples. Ceskoslovenské zdravotnictví.

[24] Bland JM, Altman DG (2000). Statistics notes. The odds ratio. BMJ (Clinical research ed).

[25] Yong Y, Lin H (2005). SHEsis, a powerful software platform for analyses of linkage disequilibrium, haplotype construction, and genetic association at polymorphism loci. Cell research.

